# To buy or not buy food online: The impact of the COVID-19 epidemic on the adoption of e-commerce in China

**DOI:** 10.1371/journal.pone.0237900

**Published:** 2020-08-20

**Authors:** Xuwen Gao, Xinjie Shi, Hongdong Guo, Yehong Liu

**Affiliations:** China Academy for Rural Development, School of Public Affairs, Zhejiang University, Hangzhou, Zhejiang, China; Shandong University of Science and Technology, CHINA

## Abstract

Drawing on a recent online survey combined with city-level data, this paper examines the impact of the COVID-19 on consumers’ online food purchase behavior in the short term. To address the potential endogeneity issues, we adopt an instrumental variable (IV) strategy, using the distance from the surveyed city to Wuhan as the instrumental variable. We show that our IV method is effective in minimizing potential bias. It is found that the share of confirmed COVID-19 cases increases the possibility of consumers purchasing food online. This is more likely to be the case for young people having a lower perceived risk of online purchases and living in large cities. Despite some limitations, this paper has policy implications for China and other countries that have been influenced by the COVID-19 epidemic. Specifically, government support and regulation should focus on (i) ensuring the safety of food sold on the internet, (ii) protecting the carrier from becoming infected, and (iii) providing financial support to the poor since they may have difficulties in obtaining access to food living in small cities. Moreover, how to help those who are unable to purchase food online because of their technical skills (e.g., the elderly who are not familiar with smart phones or the internet) also deserves more attention for the government and the public.

## Introduction

Can a disease epidemic exert a short-term effect on the development of e-commerce? While it is well understood that, with the development of e-commerce, China has become a front-runner in online retail with the largest online population growth in the world, little is known about whether and how an external shock may affect people’s online shopping choice. We examined this question in the context of a new coronavirus (COVID-19) [1, 2].

The first two decades of the new millennium have seen international concern about the outbreak of epidemics [3], with major examples including SARS (severe acute respiratory syndrome) in the early 2000s [4], Ebola in the early to mid-2010s [5], and Zika virus in the mid-2000s [6]. Currently, the start of the 2020s has been marked by a new viral respiratory pandemic (COVID-19), which is the fifth in the last 20 years and the ninth in the last century [7]. It seems that the frequency of pandemics is increasing throughout human history [8], and the public health threat from COVID-19 is one of the most serious [9].

On December 31, 2019, when 27 cases of pneumonia of unknown etiology were identified in Wuhan City, the capital of Hubei Province in China [10–13], the Chinese government first reported an outbreak of a new coronavirus disease, COVID-19 [14, 15]. The disease spread quickly to other regions. By the beginning of April, the epidemic was controlled in China through a number of effective actions, including isolated treatment, personal protective equipment, medical monitoring and sterilization. However, with the spread of the epidemic globally, the number of confirmed COVID-19 cases has exceeded 1 million.

There is a rapidly growing body of literature examining the long-term impact of a disease epidemic. Most studies focus on the long-run impact of the disease on health, indicating that there is negative relationship between early-life disease and health [16–18]. There are a few papers examining the long-term impact of the disease epidemic on other aspects. For instance, Ambrus et al. [19] examine the impact of a cholera epidemic in one neighborhood of nineteenth-century London on the urban landscape. Their results show how geographically concentrated income shocks affect the long-run spatial distribution of poverty within a city. Garenne [20] studies long-term population effect of male circumcision in HIV epidemics in sub-Saharan African. She shows no significant difference in seroprevalence in circumcised and uncircumcised groups. However, few studies have examined whether and how people influenced by an epidemic change their behaviors in the short term. One of these behaviors that may change with the outbreak of an epidemic is the adoption of e-commerce.

Recently, there is a growing literature exploring the impact of information technology (IT) adoption, which is clearly summarized in Goldfarb and Tucker [21]. Most of the studies look into the importance of socioeconomic user characteristics–such as age, gender, educational level, place of residence and income–when studying e-commerce adoption [22]. For instance, Zhu et al. [23] examine consumers’ knowledge, purchasing behavior and willingness to pay for umami seasonings (UMS) and investigate the deciding factors related to the acceptance of UMS. Many papers have also studied the factors affecting consumers’ acceptance of e-commerce, most of which focus on cultural differences and national cultural values [24–26]. In the case of China, Wang and Somogyi [27] find that ‘Chinese consumers’ attitudes and/or purchase intentions were positively linked to their perceived incentives and negatively associated with their perceived complexity for online food shopping.’ Given the context of the COVID-19 epidemic and the development of e-commerce in China [26], it is of great significance to examine whether an external shock is likely to influence e-commerce adoption, especially regarding food purchase.

To control the epidemic, the most important measure is to cut off opportunities for human-to-human transmission. The success of China's fight against the epidemic cannot be separated from the cooperation of the whole nation and citizens’ voluntary isolation at home. However, the greatest problem in this process is how to purchase food and necessities since there is a great risk of exposure to the virus when people choose to grocery shop. This, to some extent, increases the likelihood of choosing to shop online. However, the online purchase of food also entails uncertainty since China has experienced high-profile food safety scandals in the past few years that seriously challenged public confidence in the domestic food industry [28–30]. For instance, Liu et al. [28] find that meat sold online poses potential hazards, and endpoint temperature control is the most important factor to ensure the safety of meat sold online in China. There are new challenges of online purchases associated with the outbreak of the COVID-19 epidemic. On the one hand, the buyer does not know who has handled the food materials or whether the delivery personnel are likely to be infected with the virus. This perception of online purchase security risk may affect consumers’ choice. On the other hand, with the development of e-commerce in China, the logistics system in China's major cities has become very mature, but in many small cities, the distribution of fresh products still faces big challenges, which suggests that the choices regarding online shopping may present heterogeneity among different cities.

This study contributes to the literature in several ways. First, this study is one of the few studies looking into the socio-economic impacts of the COVID-19 epidemic. Second, in the context of the COVID-19 epidemic, this study conducts a unique online survey to examine whether the external shock will influence the adoption of e-commerce in the short term in China. Specifically, we investigate whether and (if so) why people are more likely to buy food on the internet. Third, the findings of this paper provide insights into support policies aiming to help those influenced by the COVID-19 in China and also offer valuable lessons for other countries that are combatting the disease. This paper is organized as follows. Section 2 presents the methods, including data, descriptive statistics and empirical strategy. Section 3 discusses the main results, and Section 4 concludes.

## Methods

### Data and descriptive statistics

**Fig 1:**
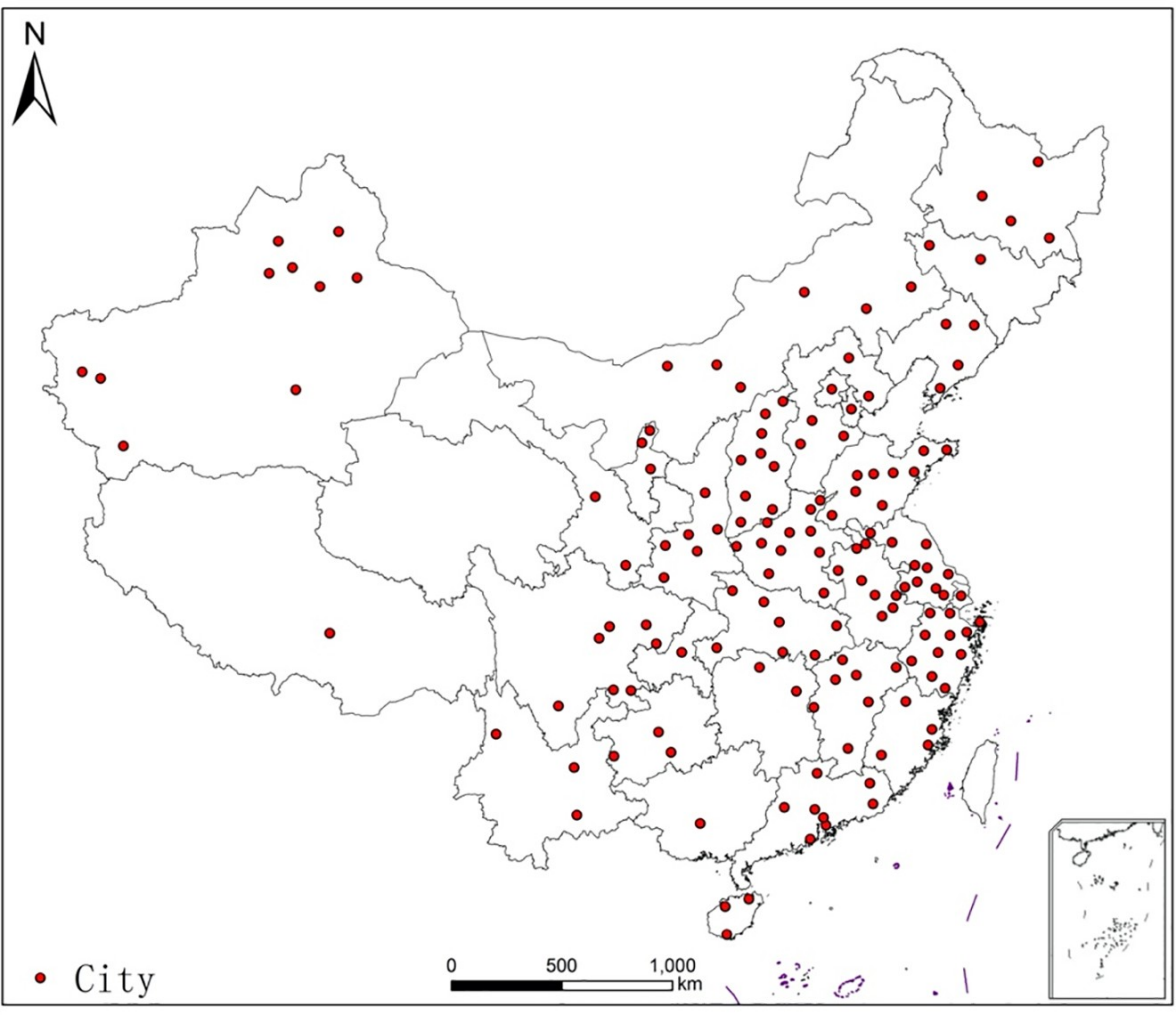
The geographical locations of the respondents.

The data used in this study are mainly from an online survey conducted through WeChat from 13 to 15 February 2020. All the participants in this study provided their informed consent to participate. Due to the influence of the COVID-19, the survey was conduected online. They were informed that if they chose to complete the questionnaire, they acquiesced in participating in this survey. The data of those who refused to complete the survey were not used in this study. All the data used in this study has been anonymized. Fig 1 shows the geographical locations of these respondents. In total, 820 individuals responded to the questionnaire; the ratio of male respondents was close to 50%, 55% of respondents were household heads, nearly half were in the age range of 19–35, and one-third were aged between 36–45. The variables also covered a variety of topics, including education, income, recent online shopping behaviors and other individual and household characteristics.

After excluding questionnaires with missing information, we are left with a sample of 770. Panel A of Table 1 shows the descriptive statistics of the main variables. Women and men are both well represented in this restricted sample. A total of 54.9% of the respondents are household heads. The average household size was close to 4. In the surveyed households, the average numbers of elderly family members and children is close to 0.7 while the maximum is 4. The education level of this sample is high: 57.8% of the respondents are college graduates, 32.3% are attending college, and only 10% have less than a college education. The age composition does not change much compared to the original sample, with 45.7% of the respondents younger than 35 years old, 31.9% aged between 36–45 and the remaining 22.3% aged 46 years old and above. With regard to the household annual income, 49% of the households earned 200,000 RMB and above in the previous year, while 29.1% of the households earned 100,000 RMB and below. It was also found that 40% of the respondents’ food was purchased from an online platform.

**Table 1:** Descriptive statistics.

	(1)	(2)	(3)	(4)	(5)
	N	mean	sd	min	max
**Household level**					
Male	770	0.497	0.500	0	1
Head	770	0.549	0.498	0	1
Household size	770	3.914	1.342	1	10
Number of old (>60)	770	0.648	0.855	0	4
Number of children (<18)	770	0.712	0.763	0	4
Dummy for college graduates	770	0.578	0.494	0	1
Dummy for college	770	0.323	0.468	0	1
Dummy for under college	770	0.0987	0.298	0	1
Age: Under 35 years old	770	0.457	0.498	0	1
Age: 36 to 45 years old	770	0.319	0.467	0	1
Age: Above 46 years old	770	0.223	0.417	0	1
HH annual income: under 100,000 RMB (Yuan)	770	0.291	0.454	0	1
HH annual income: 100,000 to 200,000 RMB (Yuan)	770	0.290	0.454	0	1
HH annual income: Above 200,000 RMB (Yuan)	770	0.419	0.494	0	1
Food expenditure from e-commerce/Food expenditure > 0.5	770	0.400	0.490	0	1
**City level**					
Population (Unit: 10,000)	150	508.2	391.0	14.80	3,391
Number of COVID-19 cases	150	107	295	0	2,791
Share of coronavirus cases (every 10,000)	150	0.206	0.525	0	3.751
Distance to Wuhan city (km)	150	960.892	716.980	56.564	3,602.756

In addition to the information from this online survey, data from the China City Statistical Yearbook were used to obtain the city population. As shown in Panel B of Table 1, there are, on average, 5.08 million people living in the surveyed cities. The number of confirmed coronavirus cases on the survey day is calculated based on official data released by the government. This figure ranges from 0 to 2791, and the average value is 107. The share of coronavirus cases is 0.00206%. To construct the instrumental variable used in this study (explained in more detail below), we also calculate the distance from the surveyed city to Wuhan city, the mean of which is 961 kilometers. Due to the strictest lockdown in Wuhan, online shopping is not possible. Thus, we drop Wuhan city in our study.

### Empirical strategy

The primary question of interest in our analysis is the causal effect of the COVID-19 shock on e-commerce, and thus, the equation of interest can be written as

$$Y_{ij}=\beta {Coro}_{j}+\alpha_{i}+\epsilon_{ij}$$ (1)

where *Y*_*ij*_ is a dummy for online food shopping after the COVID-19 epidemic for household *i* in city *j*, *Coro*_*j*_ is the number of confirmed COVID-19 cases on the survey day/city population for city *j*, and *α*_*i*_ are a list of household control variables. Estimating Eq (1) using ordinary least squares (OLS) is problematic, as the coefficients on *Coro*_*j*_ do not isolate effects due to the COVID-19 shock. COVID-19 cases and online shopping are simultaneously determined. On the one hand, online shopping could help mitigate the spread of the virus, as it reduces crowding. On the other hand, increasing COVID-19 cases forces companies to close their physical stores, which may contemporaneously affect people’s shopping. Moreover, omitted variables such as people’s average health status and the culture of the city may also bias our results. Thus, we use the distance to Wuhan city as an exogenous instrumental variable (IV) for *Coro*_*j*_, where the assumption is that the distance to Wuhan affects the choice of online shopping only through its effect on confirmed COVID-19 cases. As the center of transportation, the locked down of Wuhan rules out the possibility that closer to Wuhan would be easier to do business online, which assures the validity of our IV strategy. Another threat to our identification strategy is the small sample size which might bias the estimation of standard errors. To solve this problem, we employ the “Bootstrap” method in our analysis. Bootstrap can repeatedly draw a sample with replacement. This method is significantly helpful when the cluster size is small.

We first calculate the distance between city *j* and Wuhan using latitude and longitude coordinates. Then, the first-stage equation relating the distance to Wuhan to confirmed COVID-19 cases is

$${Coro}_{j}=\alpha {Distance}_{j}+\alpha_{j}+\epsilon_{j}$$ (2)

where *Distance*_*j*_ is the distance from city *j* to Wuhan city, and *α*_*i*_ are a list of household control variables. The standard errors are estimated using bootstrapping to correct for clustering at the city level.

Based on the average effects above, we also try to deepen our understanding of the potential heterogeneous effects. Considering the great differences in shipment access across cities, we first allow the effect of COVID-19 to vary by city administrative level by estimating:

$$Y_{ij}=\beta {Coro}_{j}*{Citylevel}_{j}+\alpha_{i}+\epsilon_{ij}$$ (3)

where *Y*_*ij*_ is still a dummy for online food shopping after the COVID-19 epidemic for household *i* in city *j*, *Citylevel*_*j*_ is an indicator for the city administrative level (city administrative levels include above prefecture-level city, prefecture-level city and below prefecture-level city).

In addition to the heterogeneity by city administrative level, various perceived risks of online shopping may affect our results. To investigate whether this is the case, we allow the effects to be specific to households’ perceived risk by replacing city-level indicators with risk indicators.

In summary, increased COVID-19 cases could, in fact, affect households’ online shopping choice, while the well-constructed IV–distance–rules out reverse causality. We further study the heterogeneous effects by dividing the cities into prefecture-level cities, prefecture-level cities and below prefecture-level cities and dividing perceived risks into high risk, moderate risk and low risk.

## Results and discussion

Based on the data and empirical strategy outlined above, this section examines the impact of coronavirus on the development of e-commerce. Starting with the OLS method, columns 1–3 in Table 2 show the preliminary results of the impact of coronavirus on online shopping behaviors. The coefficient on the share of coronavirus cases is -0.0784 and nonsignificant (Column 1 in Table 2). With the regional fixed effects included, the results barely change (Column 2 in Table 2). The introduction of provincial fixed effects leads to a nonsignificant coefficient of -0.222 (Column 3 in Table 2). With the potential endogeneity issues left aside, these results suggest that there is no link between the outbreak of COVID-19 and online shopping behaviors. However, for the reasons discussed in Section 3, the issues of reverse causality and missing variables are likely to bias the results.

**Table 2:** Effect of COVID-19 on e-commerce.

	Online shopping after the pandemic					
	(1)	(2)	(3)	(4)	(5)	(6)
	OLS	OLS	OLS	2SLS	2SLS	2SLS
Share of COVID-19 cases	-0.0784	-0.0722	-0.222	0.0535	0.134	0.704
	(0.0597)	(0.0604)	(0.149)	(0.214)	(0.302)	(0.709)
	[0.220]	[0.255]	[0.285]	[0.816]	[0.681]	[0.260]
Control variables	Yes	Yes	Yes	Yes	Yes	Yes
Regional fixed effects	No	Yes	No	No	Yes	No
Provincial fixed effects	No	No	Yes	No	No	Yes
First-stage F-stat	-	-	-	100.3	62.91	67.91
Observations	770	770	770	770	770	770

The dependent variable is a dummy for online shopping after the pandemic. The share of COVID-19 cases is calculated as the number of confirmed COVID-19 cases on the survey day/city population. The instrumental variable for the share of COVID-19 cases is the distance between the city and Wuhan, which is transformed using the log function. Control variables include gender, age, education levels, income, dummy for household head, household size, share of children and share of the elderly. The region refers to the east, center and west. Robust standard errors clustered at the city level are reported in parentheses. P-values from wild bootstrap clustering are reported in brackets. We use Rademacher weights and 1000 replications. *** significant at the 1% level; significant at the 5% level; * significant at the 10% level.

To overcome the potential endogeneity issues, we turn to the results using two-stage least squares, instrumenting the share of COVID-19 cases by the distance from the surveyed city to Wuhan. The first stage, reported in Table 3, shows that the distance has a negative impact on the share of confirmed COVID-19 cases (Column 1). The coefficient remains negative and significant when control variables, regional fixed effects and provincial fixed effects are included (Columns 2–4). This finding is consistent with that of Fan et al. [31], who found that there is a high correlation between Wuhan’s floating population and the number of confirmed cases since more than 5 million migrants living in Wuhan left Wuhan as potential carriers of the virus before the “closure of the city” and may became carriers of the virus for retransmission.

**Table 3:** Effect of distance to Wuhan on cities’ share of COVID-19 cases.

	Share of COVID-19 cases			
	(1)	(2)	(3)	(4)
Distance	-0.389***	-0.506***	-0.520***	-0.582**
	(0.127)	(0.149)	(0.167)	(0.230)
	[0.0000]	[0.0000]	[0.0000]	[0.0000]
Control variables	No	Yes	Yes	Yes
Regional fixed effects	No	No	Yes	No
Provincial fixed effects	No	No	No	Yes
Observations	150	150	150	150

The dependent variable is calculated as the number of confirmed COVID-19 cases on the survey day/city population. The distance is the distance between the city and Wuhan, which is transformed using the log function. Control variables include log(population) and city administrative level. Region refers to east, center and west. Robust standard errors clustered at the city level are reported in parentheses. P-values from wild bootstrap clustering are reported in brackets. We use Rademacher weights and 1000 replications.

*** significant at the 1% level; significant at the 5% level; * significant at the 10% level.

The coefficients on “the share of COVID-19 cases” are reported in Columns 4–6 of Table 2. If the instrumental variable is reasonable, we expect this effect to be larger than the OLS estimate, i.e., to be positive given that the OLS estimates are close to zero. We find this to be the case; the coefficient is 0.0535, as shown in Column 4, and increases to 0.134 (Column 5) and 0.704 (Column 6) when regional fixed effects and provincial fixed effects are controlled for, respectively. However, all of these coefficients are nonsignificant.

The results above seem to be inconsistent with our expectation that the outbreak of COVID-19 is likely to increase the possibility of purchasing food online. However, the average effect may be bothered by certain types of food. As a further piece of evidence, it is informative to see whether the COVID-19 epidemic encourages the consumption of high calories food under the lock-down circumstances. To better understand this phenomenon, we explore the heterogeneous effects of different food items. The estimated results are presented in Table 4. In all cases, we control for regional fixed effect. Although the coefficients of other food items remain in-significant, column 3 indicates that the outbreak of COVID-19 significantly increases the possibility of buying meat online, which is consistent with our assumption. This suggests that the purchase of high calories food–meat–is increased after the epidemic.

**Table 4:** Effect of COVID-19 on online-food purchase: By type.

	(1)	(2)	(3)	(4)	(5)
	Fruit	Vegetable	Meat	Seafood	Grain
	OLS	OLS	OLS	2SLS	2SLS
Share of COVID-19 cases	0.0109	0.390	0.409*	0.253	-0.0605
	(0.270)	(0.315)	(0.243)	(0.186)	(0.169)
	[0.970]	[0.199]	[0.056]	[0.139]	[0.726]
Control variables	Yes	Yes	Yes	Yes	Yes
Regional fixed effects	Yes	Yes	Yes	Yes	Yes
First-stage F-stat	62.91	62.91	62.91	62.91	62.91
Observations	770	770	770	770	770

The dependent variable is a dummy for online shopping by food items after the pandemic. The share of COVID-19 cases is calculated as the number of confirmed COVID-19 cases on the survey day/city population. The instrumental variable for the share of COVID-19 cases is the distance between the city and Wuhan, which is transformed using the log function. Control variables include gender, age, education levels, income, a dummy for household head, household size, the share of children and share of the elderly. The region refers to the east, center and west. Robust standard errors clustered at the city level are reported in parentheses. P-values from wild bootstrap clustering are reported in brackets. We use Rademacher weights and 1000 replications. *** significant at the 1% level; significant at the 5% level

* significant at the 10% level.

Furthermore, COVID-19 epidemic might have a differential effect on certain socioeconomic groups of the population. One of these factors is obviously that people living in small or large cities may have different access to logistics services. To address this issue, we add interaction terms between the share of confirmed cases and the city scale. The results are shown in Table 5, where the above prefecture-level cities include sub-provincial and provincial cities, and below prefecture-level cities include counties and below. It is found that the coefficients on the interaction terms between the share of confirmed cases and the dummy of below prefecture-level city are nonsignificant regardless of the estimation method used, either OLS and 2SLS. However, with regard to the interaction terms between the share of confirmed cases and the dummy of (above) prefecture-level city, we find that people in larger cities are more likely to shop online after the outbreak of the COVID-19 epidemic.

**Table 5:** Heterogeneous effect of COVID-19 on e-commerce: By city administrative level.

	Online shopping after the pandemic		
	(1)	(2)	(3)
	2SLS	2SLS	2SLS
Above prefecture-level city * share	1.062***	1.224***	1.612**
	(0.308)	(0.352)	(0.741)
	0.00100	0.00100	0.0260
Prefecture-level city * share	0.425	0.650*	1.127*
	(0.344)	(0.436)	(0.730)
	0.182	0.0880	0.0800
Below prefecture-level city * share	-0.297	-0.172	0.352
	(0.247)	(0.294)	(0.669)
	0.148	0.578	0.563
Control variables	Yes	Yes	Yes
Regional fixed effects	No	Yes	No
Provincial fixed effects	No	No	Yes
First-stage F-stat	28.91	17.52	20.38
Observations	770	770	770

The dependent variable is a dummy for online shopping after the pandemic. The above prefecture-level cities include sub-provincial and provincial cities. Below prefecture-level cities includes counties and below. The share of COVID-19 cases is calculated as the number of confirmed COVID-19 cases on the survey day/city population. The instrumental variable for the share of COVID-19 cases is the distance between the city and Wuhan, which is transformed using the log function. Control variables include gender, age, education levels, income, dummy for the household head, household size, share of children and share of elderly. The region refers to the east, center and west. Robust standard errors clustered at the city level are reported in parentheses. P-values from wild bootstrap clustering are reported in brackets. We use Rademacher weights and 1000 replications.

*** significant at the 1% level; significant at the 5% level

* significant at the 10% level.

In S1 Table, we replace the dependent variable with a dummy indicating whether online food expenditure exceeds 50 percent of household food expenditure, further verifying that, with the increase in the number of confirmed cases, people living in larger cities are likely to buy more food online. This provides additional evidence on the robustness of our results.

The different shopping choices between the people living in large cities and those in small cities may stem from the logistics system. Large Chinese cities, such as Shanghai and Hangzhou, are centers of shipping and manufacturing, and e-commerce activities are very active with many delivery couriers. In contrast, in some of the small cities, towns and rural areas, the courier service is just starting. To examine how this is associated with people’s online shopping activities, we build a similar model to that in Table 5 and further include interaction terms between city scale, share of confirmed cases, and a dummy indicating whether the main reason for not buying food online is living out of delivery range. The results in Table 6 show that people living out of delivery range are less likely to purchase food online (mainly because they are unable to). Together with the results in Table 5, these findings suggest that people in large cities are less likely to be influenced by the epidemic since the fewer constraints on the logistics system enable them to buy food (and other living items) online.

**Table 6:** Reason for the heterogeneous effect of COVID-19 on e-commerce: By city level.

	Online shopping after the pandemic		
	(1)	(2)	(3)
	2SLS	2SLS	2SLS
Above prefecture-level city * share	3.880***	4.300***	5.733**
	(0.545)	(0.625)	(1.317)
	[0.000]	[0.000]	[0.016]
Prefecture-level city * share	4.506***	5.166***	6.084***
	(1.019)	(1.147)	(1.499)
	[0.000]	[0.000]	[0.006]
Below prefecture-level city * share	3.456***	3.804***	5.102***
	(0.734)	(0.796)	(1.250)
	[0.000]	[0.000]	[0.001]
Above prefecture-level city * share * unable to deliver	-4.429***	-4.553***	-4.509***
	(0.326)	(0.355)	(0.407)
	[0.000]	[0.000]	[0.000]
Prefecture-level city * share * unable to deliver	-5.207***	-5.389***	-5.423***
	(0.957)	(1.014)	(0.913)
	[0.000]	[0.000]	[0.000]
Below prefecture-level city * share * unable to deliver	-3.954***	-3.975***	-4.058***
	(0.656)	(0.673)	(0.678)
	[0.000]	[0.000]	[0.000]
Control variables	Yes	Yes	Yes
Regional fixed effects	No	Yes	No
Provincial fixed effects	No	No	Yes
Observations	770	770	770

The dependent variable is a dummy for online shopping after the pandemic. The above prefecture-level cities include sub-provincial and provincial cities. Below prefecture-level cities includes counties and below. The share of COVID-19 cases is calculated as the number of confirmed COVID-19 cases on the survey day/city population. The unable to deliver variable is a dummy for living out of delivery range. The instrumental variable for the share of COVID-19 cases is the distance between the city and Wuhan, which is transformed using the log function. Control variables include gender, age, education levels, income, dummy for household head, household size, share of children and share of elderly. The region refers to the east, center and west. Robust standard errors clustered at the city level are reported in parentheses. P-values from wild bootstrap clustering are reported in brackets. We use Rademacher weights and 1000 replications.

*** significant at the 1% level; significant at the 5% level; * significant at the 10% level.

Teo and Liu [32] showed that consumers’ perceived risk is negatively related to their trust in e-commerce. Under the circumstance of the COVID-19 epidemic, the impact of COVID-19 on people’s decision to buy food online may be moderated by people’s subjective awareness of the risk of shopping online in a similar way. While much online shopping is safe and reliable due to the development of e-commerce in China, many people are still concerned about the high risk of being infected when buying food online since they do not know who packaged and touched their food and whether the courier was infected by the virus. In this way, those who perceive that there is a high risk of being infected by shopping online are less likely to shop online after the pandemic given the increasing number of confirmed COVID-19 cases.

To examine whether this is the case, we regress the online shopping choice on the interactions between the share of confirmed COVID-19 cases and the dummies for the perceived risk level of becoming infected through online shopping. Note that the share of coronavirus cases is instrumented by the logarithmic form of the distance between the surveyed city and Wuhan. In the first row of Table 7, it is found that the impact of confirmed COVID-19 cases is moderated by the high-risk dummy: those who perceive a high risk of becoming infected through online shopping are less likely to buy food online. This is further confirmed by the results in the third row, showing that with a higher number of confirmed COVID-19 cases, those who pay less attention to the risk are more likely to buy food online.

**Table 7:** Heterogeneous effects of COVID-19 on e-commerce: By perceived risk.

	Online shopping after the pandemic		
	(1)	(2)	(3)
	2SLS	2SLS	2SLS
High risk* share	-1.303***	-1.197**	-0.593
	(0.429)	(0.538)	(0.932)
	[0.00800]	[0.0210]	[0.445]
Moderate risk * share	-0.359	-0.261	0.360
	(0.258)	(0.316)	(0.749)
	[0.121]	[0.345]	[0.582]
Low risk * share	0.606**	0.702**	1.258*
	(0.297)	(0.386)	(0.825)
	[0.0170]	[0.0300]	[0.0870]
Control variables	Yes	Yes	Yes
Regional fixed effects	No	Yes	No
Provincial fixed effects	No	No	Yes
First-stage F-stat	33.93	20.83	22.21
Observations	770	770	770

The dependent variable is a dummy for online shopping after the pandemic. The perceived risk level is operationalized by asking, what do you think about the risk of becoming infected through online shopping? The share of COVID-19 cases is calculated as the number of confirmed COVID-19 cases on the survey day/city population. The instrumental variable for the share of COVID-19 cases is the distance between the city and Wuhan, which is transformed using the log function. Control variables include gender, age, education levels, income, dummy for household head, household size, share of children and share of elderly. The region refers to the east, center and west. Robust standard errors clustered at the city level are reported in parentheses. P-values from wild bootstrap clustering are reported in brackets. We use Rademacher weights and 1000 replications.

*** significant at the 1% level; significant at the 5% level; * significant at the 10% level.

To ensure the robustness of this result, we replace the dependent variable with a dummy indicating whether online food expenditure accounts for more than 50 percent of the total household food expenditure. The results in S2 Table confirm that, with the increase in the number of confirmed cases, those who perceive a low risk of online purchase are more likely to purchase more food online.

In addition to the variation by city size and subjective perceived risk of online purchase, the impact of COVID-19 on consumers’ online shopping choice could vary between the young and the elderly. Hernandez et al. [33] find that socioeconomic variables–including age–moderate neither the influence of previous use of the internet nor the perceptions of e-commerce, which suggests that these variables do not condition the behavior of the experienced e-shopper. In contrast to their results, our results, shown in Table 8, suggest that in the context of COVID-19, the impact of the share of confirmed cases on online purchase choice is moderated by age; with the increase in confirmed COVID-19 cases, young people are more likely to purchase food online than elderly people. We also test whether this is still the case when we use the dummy indicating whether more than 50 percent of the household’s food is purchased online as the dependent variable. The results in S3 Table show findings similar to those in Table 8.

**Table 8:** Heterogeneous effects of COVID-19 on e-commerce: By age of the household head.

	Online shopping after the pandemic		
	(1)	(2)	(3)
	2SLS	2SLS	2SLS
Share of coronavirus cases	0.010	0.097	0.824
	(0.221)	(0.301)	(0.772)
	0.969	0.748	0.227
Share of coronavirus cases * household head age below 35	0.547***	0.469**	0.512**
	(0.211)	(0.206)	(0.223)
	[0.008]	[0.021]	[0.018]
Control variables	Yes	Yes	Yes
Regional fixed effects	No	Yes	No
Provincial fixed effects	No	No	Yes
First-stage F-stat	50.84	32.82	33.46
Observations	770	770	770
Test Share of coronavirus cases + Share of coronavirus cases * household head age below 35 = 0	0.557*	0.566*	1.336*
	[0.051]	[0.076]	[0.077]

The dependent variable is a dummy for online shopping after the pandemic. Household head age below 35 is a dummy for household head age less than 35. Above prefecture-level cities include sub-provincial and provincial cities. Below prefecture-level cities include counties and below. The share of COVID-19 cases is calculated as the number of confirmed COVID-19 cases on the survey day/city population. The instrumental variable for the share of COVID-19 cases is the distance between the city and Wuhan, which is transformed using the log function. Control variables include gender, age, education levels, income, household size, share of children and share of the elderly. The region refers to the east, center and west. Robust standard errors clustered at the city level are reported in parentheses. P-values from wild bootstrap clustering are reported in brackets. We use Rademacher weights and 1000 replications.

*** significant at the 1% level

** significant at the 5% level

* significant at the 10% level.

## Conclusions

COVID-19 is more than a worldwide public health emergency; it is an international economic crisis that could surpass the global financial crisis of 2008–09 [34]. Drawing on a recent online survey combined with provincial-level data, this paper examines the impact of COVID-19 on consumers’ online food purchase behavior in the short term. To address the potential endogeneity issues, we adopt an IV strategy, using the distance from the surveyed city to Wuhan as the instrumental variable. We find that the share of confirmed COVID-19 cases increases the possibility of consumers purchasing food online. This is more likely to be the case for young people having a lower perceived risk of online purchase and living in large cities.

Some limitations of this paper should be noted since they may have implications for future research. First, the main data used in this study are from an online survey since the focus of this study is on the short-term impact, which underlines the need to perform a longitudinal analysis if we seek to better understand the long-term impact. Second, it must be remembered that this study was undertaken in China, and whether the results of this paper are applicable to other countries remains uncertain. With the spread of the COVID-19 epidemic globally, it would be of great significance to examine whether and how the results differ in other countries. Third, our results suggest that the outbreak of the COVID-19 epidemic increases the possibility of purchasing food online, but we do not discuss whether there is reverse causality in that consumers’ choice of purchasing food online is likely to impact epidemic prevention and control. This lies beyond the scope of this paper.

Keeping these limitations in mind, our paper has some policy implications for China and other countries. First, since it is found that consumers are more likely to purchase food online due to the COVID-19 epidemic, government support and regulation should focus on ensuring the safety of food sold on the internet. More attention should also be given to protecting the carrier from becoming infected since there is a possibility of human-to-human transmission of the virus. Second, since our paper also shows that young people living in large cities are more likely to purchase food online, the government should provide financial support to the poor since they may have difficulties in obtaining access to food living in small cities. Moreover, how to help those who are unable to purchase food online because of their technical skills (e.g., the elderly who are not familiar with smart phones or the internet) also deserves more attention for the government and the public.

## Supporting information

S1 Data (DTA)

S1 Table Heterogeneous effect of COVID-19 on the share of online food expenditure: By city administrative level. (DOCX)

S2 Table Heterogeneous effect of COVID-19 on the share of online food expenditure: By perceived risk. (DOCX)

S3 Table Heterogeneous effect of COVID-19 on the share of online food expenditure: By household head age. (DOCX)
