# Supplementary material for: To buy or not buy food online: The impact of the COVID-19 epidemic on the adoption of e-commerce in China
Source: PLoS One. 2020 Aug 20;15(8):e0237900. doi: 10.1371/journal.pone.0237900 (PMC7440641; doi:10.1371/journal.pone.0237900)
Supplement: S1 Table — (DOCX) [file pone.0237900.s002.docx]

**S1 Table: Heterogeneous effect of COVID-19 on the share of online food expenditure: by city administrative level.**

|  | **High share of e-commerce food expenditure** | | |
| --- | --- | --- | --- |
|  | **(1)** | **(2)** | **(3)** |
|  | **2SLS** | **2SLS** | **2SLS** |
| Above prefecture-level city * share | 1.376*** | 1.543*** | 1.547** |
|  | (0.307) | (0.355) | (0.688) |
|  | [0.000] | [0.000] | [0.026] |
| Prefecture-level city * share | 0.609* | 0.841** | 0.820 |
|  | (0.367) | (0.459) | (0.679) |
|  | [0.066] | [0.037] | [0.209] |
| Below prefecture-level city * share | 0.004 | 0.134 | 0.255 |
|  | (0.220) | (0.271) | (0.590) |
|  | [0.988] | [0.598] | [0.634] |
| Control variables | Yes | Yes | Yes |
| Regional fixed effects | No | Yes | No |
| Provincial fixed effects | No | No | Yes |
| Observations | 770 | 770 | 770 |

*Notes:* The dependent variable is a dummy variable for online food expenditure exceeding 50 percent of household food expenditure. The above prefecture-level cities include sub-provincial and provincial cities. Below prefecture-level cities includes counties and below. The share of COVID-19 cases is calculated as the number of confirmed COVID-19 cases on the survey day/city population. The instrumental variable for the share of COVID-19 cases is the distance between the city and Wuhan, which is transformed using the log function. Control variables include gender, age, education level, income, dummy for household head, household size, share of children and share of the elderly. The region refers to the east, center and west. Robust standard errors clustered at the city level are reported in parentheses. P-values from wild bootstrap clustering are reported in brackets. We use Rademacher weights and 1000 replications. *** significant at the 1% level; significant at the 5% level; * significant at the 10% level.
