# Supplementary material for: To buy or not buy food online: The impact of the COVID-19 epidemic on the adoption of e-commerce in China
Source: PLoS One. 2020 Aug 20;15(8):e0237900. doi: 10.1371/journal.pone.0237900 (PMC7440641; doi:10.1371/journal.pone.0237900)
Supplement: S3 Table — (DOCX) [file pone.0237900.s004.docx]

**S3 Table: Heterogeneous effect of COVID-19 on the share of online food expenditure: by household head age**

|  | **High share of e-commerce food expenditure** | | |
| --- | --- | --- | --- |
|  | **(1)** | **(2)** | **(3)** |
|  | **2SLS** | **2SLS** | **2SLS** |
| Share of coronavirus cases | 0.291 | 0.383 | 0.802 |
|  | (0.241) | (0.316) | (0.781) |
|  | [0.191] | [0.181] | [0.262] |
| Share of coronavirus cases * household head age below 35 | 0.555*** | 0.475** | 0.527*** |
|  | (0.205) | (0.199) | (0.203) |
|  | [0.007] | [0.013] | [0.016] |
| Control variables | Yes | Yes | Yes |
| Regional fixed effects | No | Yes | No |
| Provincial fixed effects | No | No | Yes |
| Observations | 770 | 770 | 770 |
| Test Share of coronavirus cases + Share of coronavirus cases * household head age below 35 = 0 | 0.856*** | 0.858** | 1.329* |
|  | [0.006] | [0.020] | [0.069] |

*Notes:* The dependent variable is a dummy variable for online food expenditure exceeding 50 percent of household food expenditure. Household head age below 35 is a dummy for household heads aged under 35. The above prefecture-level cities include sub-provincial and provincial cities. Below prefecture-level cities include counties and below. The share of COVID-19 cases is calculated as the number of confirmed COVID-19 cases on the survey day/city population. The instrumental variable for the share of COVID-19 cases is the distance between the city and Wuhan, which is transformed using the log function. Control variables include gender, age, education level, income, household size, share of children and share of elderly. The region refers to the east, center and west. Robust standard errors clustered at the city level are reported in parentheses. P-values from wild bootstrap clustering are reported in brackets. We use Rademacher weights and 1000 replications. *** significant at the 1% level; significant at the 5% level; * significant at the 10% level.
